# Poisoning by a Small Dose of the Muriate of Morphia Applied Externally

**Published:** 1845-11

**Authors:** 


*Poisoning by a Small Dose of the Muriate of Morphia applied Externally.*—A young woman, laboring under scirrhus of the uterus, and suffering from vomiting and pain in the stomach, was ordered to apply to the epigastrium, from which the skin had been previously removed by a blister, the 1-32nd part of a grain of the muriate of morphia. The same dose was repeated by the endermic method the following morning. Some time afterwards, the woman fell into a state of complete narcotism. She suffered from pain in the head, stupor, ringing in the ears, dizziness, and incoherency, a hot and dry skin, and a strong and frequent pulse. Among the symptoms was one somewhat remarkable—namely, that she saw only the half of surrounding objects; for instance, in the case of a person standing before her, she could only see the right or left half of the body. The cerebral congestion was followed by convulsions. Venesection was performed, but this only produced a stronger attack, followed by another. A compress, soaked in vinegar, with ice, was applied to the forehead, followed by mustard poultices to the lower extremities. The symptoms gradually abated, but it was three weeks before vision and speech were perfectly restored.—*London Medical Gazette.*

